# Forbearing One Another

**Published:** 1867-10

**Authors:** 


### HALL'S JOURNAL OF HEALTH.

**Our Legitimate Scope is almost boundless; for whatever begets pleasurable and harmless feelings, promotes Health; and whatever induces disagreeable sensations, engenders Disease.**

WE AIM TO SHOW HOW DISEASE MAY BE AVOIDED, AND THAT IT IS BEST, WHEN SICKNESS COMES, TO TAKE NO MEDICINE WITHOUT CONSULTING A PHYSICIAN.

VOL. XIV.] OCTOBER, 1867. [No. 10.

### FORBEARING ONE ANOTHER.

"FORBEARING one another in love" is a Bible injunction, and if carried out practically in every department of human life as between neighbors, friends, fellow citizens; as between employers and employed; master and servant; brother and sister; parent and children; husband and wife; minister and people, governor and governed,— one-half of all the misery of human life would be averted; millions would be prevented from giving themselves up to habitual crime; other millions would be prevented from being discouraged into thriftlessness and degrading indulgences, and a multitude which no man can number would be invited upwards to fill positions of usefulness, trust and honor, who otherwise will die by violence, in prisons, or under the gallows.

A gentleman, now a New York millionaire, married a beautiful widow his second wife; they had been mutually acquainted some years. By some means the husband was detained to a late hour in the night, within a week after their marriage; the new wife was found waiting for him; the door being locked she was asked to open it; the request was denied; the husband quietly repaired to another room, and at the end of twelve years has never visited her, nor ever will, most likely.

A young wife, within a few months after her marriage, was horrified one night on going to the door to let her husband in, to find him intoxicated—so much so that he could hardly stand. A thunder-stroke in a clear winter's sky could not have more surprised her; friends were in the drawing-room, expecting him every moment; with a woman's quickness and instinct, she hurried him to his chamber, with loving words put him to sleep, made some excuse for the mutual benefit of friends and servants, watched over him through the weary hours of night, and next morning when he waked, she had no harsh reproof ready for him, no withering rebuke, but tenderly alluded to the facts of the case, and kissed away his confusion; there it dropped; he never tasted another drop; raised a family of sons who are respectable and successful merchants in New York and Philadelphia, and dying, left a name for mercantile integrity which any man might envy.

But the principles may be illustrated with other recitals than our own.

A merchant in London had a dispute with a Quaker respecting the settlement of an account. The merchant was determined to bring the account into court, a proceeding the Quaker earnestly deprecated, using every argument in his power to convince the merchant of his error; but the latter was inflexible. Desirous to make a last effort, the Quaker called at his house one morning, and inquired of the servant if his master was at home. The merchant hearing the inquiry, and knowing the voice, called out from the top of the stairs, "Tell that rascal I am not at home." The Quaker, looking up to him, calmly said, "Well, friend, God put thee in a better mind."

The merchant, struck afterward with the meekness of the reply, and having more deliberately investigated the matter, became convinced that the Quaker was right and he was wrong.

He requested to see him, and after acknowledging his error, he said: "I have one question to ask you; how were you able, with such patience, on various occasions to hear my abuse?"

"Friend," replied the Quaker, "I will tell thee: I was naturally as hot and violent as thou art. I knew that to indulge this temper was sinful, and I found it was imprudent. I found that men in a passion always spake loud; and I thought if I controlled my voice I should repress my passion. I have, therefore, made it a rule never to let my voice rise above a certain key, and by a careful observation of this rule, I have, by the blessing of God, entirely mastered my natural temper."

The Quaker reasoned philosophically, and the merchant, as every one else may do, benefitted by his example.

Says another writer: —

Who has not observed, in every relation of life, harmony and good feelings depend on not pushing things to extremities, not pressing rights to the uttermost, not contending to death for trifles? Whether the relation be that of parents and children, masters and servants, partners in business, or councillors or directors of public business, it is forbearance that oils the wheels and enables the machinery to work smoothly, and at the same time efficiently. Of course "forbearing," like "bearing," must have its limits. But no small point is gained when the necessity of this quality in some form and to some extent, is apprehended by all; when people, and especially young people, come to see if they are to get on comfortably with their fellows, there must be some forbearance in expressing their opinions, or even urging their rights; there must be some consideration, even for the infirmities and unreasonableness of others—some care taken to not drive them, without cause, into open hostility—not to bring things to a dead lock with them. It is melancholy to think what feuds have been waged through want of this forbearance.

The following beautiful narration is from that very excelent religious newspaper, the CHRISTIAN WATCHMAN AND REFLECTOR, entitled:

